# The Use of Dried Matrix Spots as an Alternative Sampling Technique for Monitoring Neglected Tropical Diseases

**DOI:** 10.3390/pathogens13090734

**Published:** 2024-08-29

**Authors:** Wanesa Richert, Krzysztof Korzeniewski

**Affiliations:** Department of Epidemiology and Tropical Medicine, Military Institute of Medicine-National Research Institute, 128 Szaserów St., 04-141 Warsaw, Poland; wrichert@wim.mil.pl

**Keywords:** dried matrix spots, neglected tropical diseases, diagnostics

## Abstract

Neglected tropical diseases (NTDs) are a group of illnesses which usually present with a chronic clinical picture. NTDs can lead to permanent disability and are often associated with social stigma. In many developing countries where NTDs are endemic, there are no diagnostic tools for the safe storage and transport of biological samples, and there are no specialist diagnostic centers where the samples could be processed. The transport of biological samples (blood, urine) collected in field conditions and brought to laboratories located in developed countries requires the maintenance of the cold chain during transportation. Ensuring temperature control during transport could be problematic or even impossible to achieve; it is also expensive. A helpful solution to this problem is to use the dried matrix spot (DMS) technique, which seems to be a reliable method for collecting biological samples to be used for screening purposes and conducting epidemiological surveillance of NTDs in developing countries. This article is an overview of how DMSs can be used in the diagnosis of most neglected tropical diseases.

Neglected tropical diseases (NTDs) are a group of illnesses caused by various etiological factors, e.g., bacteria, fungi, viruses and parasites. Most NTDs are chronic and debilitating conditions which can lead to permanent disability and are often associated with social stigma or exclusion. Some NTDs have a long incubation period and therefore can be difficult to diagnose [1]. NTDs are primarily prevalent in low-income, tropical or subtropical countries. Their occurrence is determined by poor sanitation, regular contact with reservoirs of infections (infected people or animals) and limited access to healthcare. Climate change and population growth facilitate the spread of NTDs, but global eradication initiatives still prioritize the diagnosis and treatment of AIDS, malaria and tuberculosis rather than NTDs. In 2021, the World Health Organization (WHO) initiated a global project titled Ending the neglect to achieve the Sustainable Development Goals: a road map for neglected tropical diseases 2021–2030, which sets out goals for the prevention, control and elimination of NTDs worldwide. Despite these efforts, NTDs remain a serious health issue in many countries globally, especially in neglected communities living in extreme poverty. Every year, NTDs are responsible for 200,000 deaths globally. People affected by NTDs are not only at risk of various disabilities, disfigurement and social stigma, but they are also in danger of socio-economic exclusion because they are unfit to work. In addition, the treatment of NTDs puts considerable strain on family budgets in many developing countries [2,3,4,5]. According to the World Health Organization, NTDs include 20 diseases and disorders: Buruli ulcer, Chagas disease, dengue and chikungunya, dracunculiasis, echinococcosis, foodborne trematodiases, human African trypanosomiasis, leishmaniasis, leprosy, lymphatic filariasis, mycetoma, chromoblastomycosis and other deep mycoses, onchocerciasis, rabies, scabies and other ectoparasitoses, schistosomiasis, snakebite envenoming, soil-transmitted helminthiases, taeniasis and cysticercosis, trachoma and yaws. NTDs are difficult to control because many of them are vector-borne illnesses that are transmitted from infected animals and are often caused by pathogenic organisms which have complex life cycles [6].

NTDs can be eradicated by promoting health education, personal hygiene, the use of insect repellents, proper sanitation, immunization and treatment. However, it is equally important to focus of the diagnostics of NTDs and to develop and implement workable solutions for the detection of pathogens responsible for causing these conditions [4,7].

In countries with limited diagnostic capabilities, even the first diagnostic stage (i.e., the collection, processing, transport and storage of biological samples) can be extremely problematic [8]. A helpful solution to this problem could be the application of the increasingly popular dried matrix spot (DMS) sampling technique. This technique consists of applying a small amount of a liquid biological sample, such as blood, urine, saliva, sweat, cerebrospinal fluid, etc., onto specially manufactured filter paper and leaving it to dry [9,10]. The dried matrix spots can be used in bioanalysis using a range of tools and techniques, including chromatography, mass spectrometry, DNA analysis and immunoenzymatic tests [11]. This means, that the DMS technique could successfully be used for multiple purposes, including the surveillance of illnesses caused by microbiological agents, genetic testing, drug monitoring, clinical pharmacotherapy, forensic toxicology or environmental contamination control [12,13,14,15,16,17]. DMS testing dates back to 1963, when Guthrie and Susi [18] developed an assay for the detection of phenylketonuria in neonates. For this purpose, they collected capillary blood samples from neonates using the heel prick method, applied the samples onto filter paper, left the samples to dry, and then used the dried blood spots to measure the level of phenylalanine. This breakthrough invention gave rise to the diagnosis of many other congenital and inherited disorders and led to the introduction of large-scale newborn screening programs [19]. It also proved effective in the diagnosis of many infectious diseases such as syphilis, trypanosomiasis, amoebiasis, rubella and hepatitis B [20,21,22,23]. Over the next few decades, there was an increase in interest in the use of DBSs, and thanks to the development of this and other novel diagnostic techniques, it was possible to improve accessibility to diagnostics even in the most remote areas of the world [24].

DMS sampling is a suitable alternative to traditional sampling methods, such as the collection of wet plasma and serum samples, especially in settings with limited diagnostic capabilities or shortages of qualified personnel. This technique is also a helpful solution in situations when the transport of liquid biological samples would be problematic. DMS samples, even if collected outside healthcare facilities, are a good alternative to rapid diagnostic tests (RDTs) [25].

Another advantage of this diagnostic method is the small sample size, which contributes to higher analyte stability. In addition, DMS sampling is cost-effective, as dried specimens are easy to store. Processing DMSs is also safer because it is associated with a much lower risk of transmitting an infection (the process of drying damages the envelope of some viruses and can reduce their infectivity). The transportation of dried sample matrices is also much safer compared to the transport of liquid samples, as there is no risk of damage to transport containers or leakage of samples. Another advantage of this technique is the fact that there is no need for centrifugation to separate serum from blood clots, which further limits the risk of exposure to potentially infectious material [26,27].

As was mentioned before, a small volume of the sample helps stabilize the analyte but is associated with potentially lower analyte concentration. For this reason, DMS testing requires the use of more sensitive analytical tools and techniques [9,28]. A lower concentration of the analyte is correlated with lower analytical sensitivity of the assays performed on DBSs compared to tests on serum/plasma or other liquid samples (biomarker concentrations can be low during an infection), but the analytical sensitivity of the DBS technique generally exceeds the analytical sensitivity of RDTs [25]. The pre-analysis of DMS samples is performed manually and it involves cutting out a disc of a selected diameter from the filter paper and placing the disc in a test tube filled with appropriate buffer solution and eluting it for a minimum of 2 h on a shaker. All these procedures require rigorous validation in order to ensure reliable test results [14,25]. Another positive feature of the DMS samples is their long-term stability. Obviously, analyte stability can be affected by factors such as the type of filter paper used for sample collection, exposure of the specimen to sunlight, the temperature or humidity and the type of the target analyte. Nevertheless, if dried matrices are stored properly, they retain their properties for a long time and can be used for clinical testing for up to several years [29]. The DMS method has certain limitations, of which the lack of standardization of the pre-analytical phase is one of the most important. Only the DBS tests for newborn screening are conducted in line with the approved preparatory protocol, whereas no such protocols exist for any other DBS tests. Depending on the type and the amount of biological material used for testing, as well as the type of filter paper and the method of DMS extraction, the analytical efficiency may vary significantly between different tests. One should also bear in mind that hemolysis may occur while applying a blood specimen onto a filter paper, and this may give a false negative result in some cases. These limitations require careful pre-evaluation and refining of the test’s methodology [29]. However, the sensitivity and specificity of DBSs is higher than that of RDTs, which allows for more precise testing and accurate results [25]. A major disadvantage of DBSs, in comparison to RDTs, is the length of the diagnostic procedure (it takes longer to obtain a result) and the need to maintain appropriate microbiological purity, which is a serious obstacle in field-testing.

The aim of the present article is to demonstrate an alternative method for the collection of specimens used in the diagnosis of neglected tropical diseases, whose application could greatly improve the health of thousands of people affected by extreme poverty and exclusion. For this purpose, the authors searched the electronic database PubMed for observational studies and randomized controlled trials on diagnosing NTDs. We only focused on those reports in which the use of DMSs had a positive impact on the diagnostic results.

There are numerous reports in the literature on DMSs being used for the diagnosis of NTDs. As an example, DBSs can be used to perform serological tests for the diagnosis of echinococcosis [29,30,31,32,33], Chagas disease [34,35,36,37,38], dengue and chikungunya viruses [39,40,41,42,43,44], foodborne trematodiases [45,46,47], human African trypanosomiasis [48,49,50,51,52], leishmaniasis [8,53,54,55], leprosy [56,57], lymphatic filariasis [58,59,60,61,62], onchocerciasis [63,64], schistosomiasis [65,66,67], trachoma [68,69,70,71,72], yaws [73,74], taeniasis and cysticercosis [75,76,77,78], as well as soil-transmitted helminthiases [79,80]. Dried urine spots (DUS) are used for the diagnosis of the circulating cathodic antigen (CCA) of *Schistosoma mansoni* [81,82], dried saliva spots (DSS) are used for the serodiagnosis of the dengue virus [39], and dried cerebrospinal fluid (CSF) is used in ELISA tests for cysticercosis [83]. Dried blood samples collected from foxes, dogs and racoon dogs are commonly used for the serodiagnosis of rabies [84,85]. Dried matrix spots have also been found to be effective in molecular diagnostics. Loop-mediated isothermal amplification (LAMP) assays are capable of detecting Chagas disease [86] and leishmaniasis [87,88] from DBS samples, and the LAMP method is also effective in diagnosing schistosomiasis from DUS samples [89]. Quantitative real-time PCR (qPCR) assays using DMSs can be used to diagnose dengue and chikungunya viruses [90], Buruli ulcer [91] and leishmaniasis [8,92]. According to the literature, gel-based PCR is the most common diagnostic method for the detection of NTDs from dried matrix spots. This technique is effective in diagnosing Chagas disease [34,93], lymphatic filariasis [94,95], dengue virus infection [96,97,98,99], human African trypanosomiasis [100], leishmaniasis [101,102,103,104,105], onchocerciasis [62] and schistosomiasis [106,107,108,109,110]. Rabies virus can be detected with RT-PCR assays in DBS samples collected from infected dogs or with reverse transcription followed by a hemi-nested polymerase chain reaction (RT-hn-PCR), and in the case of wild animals, in dried brain tissue samples stored on filter paper [111,112]. There are reports in the literature which support the validity of using FTA cards for the diagnosis of mycetoma, chromoblastomycosis and other deep mycoses, and study results suggest that both serological and molecular methods are effective in diagnosing mycoses; however, this issue requires further research. Table 1 shows the diagnostic possibilities of DMSs for the diagnosis of NTDs.

## Summary

Limited access to specialist diagnostic facilities in countries where NTDs are endemic is a major restraint for the safe storage and transport of biological samples. The transport of biological samples collected in field conditions and brought to laboratories located in developed countries requires the maintenance of the cold chain during transportation. Ensuring temperature control during transport could be problematic or even impossible to achieve, and it is also expensive. A good solution to this problem is to use the dried matrix spot (DMS) technique, which is a reliable method for collecting biological samples to be used for the diagnosis and epidemiological surveillance of NTDs. It needs to be emphasized that the DBS or DUS sampling technique will never replace tests on wet plasma, serum or urine matrices; however, following careful test validation to ensure its high sensitivity and specificity, the DMS technique could become a reliable testing method for the diagnosis of most NTDs, as evidenced by this review. Given the fact that many tropical illnesses are co-endemic in certain areas, it would be possible to monitor several diseases affecting a given community simultaneously simply by using the existing infrastructure and non-invasive DMS sampling method. This intervention could simplify the process of the epidemiological surveillance of NTDs, reduce the costs of NTD monitoring, and help control outbreaks of existing and emerging illnesses, especially in low-income, tropical countries.

The present review summarizes the DMS method, which has successfully been used in the diagnosis of NTDs in recent years, despite the fact that there are few publications available on DMS sample preparation and validation. The review provides a solution for those medical diagnostic centers which are located far from the areas affected by NTDs, where the collection and safe transport of samples is a challenge. This convenient, easy and relatively inexpensive sampling method represents an important advancement in medical research, especially in hard-to-reach populations, in populations without access to healthcare or in those heavily dependent on external support. One of the most important problems encountered by the authors while searching for relevant publications was the lack of standardization of the methodology and sample validation. For this reason, the results reported by different authors were not uniform or comparable. Although the DMS technique represents a promising sampling alternative which could be used in remote areas affected by extreme poverty, it requires refinement and the development of a uniform methodology.

## Figures and Tables

**Table 1:** The use of dried matrix spots in the diagnostics of NTDs.

Disease	Material	Diagnostic Assay	Reference
Buruli ulcer	DBS	qPCR	[91]
Echinococcosis	DBS	immunoenzymatic assay	[30,31,32,33]
Chagas disease	DBS	immunoenzymatic assay	[34,35,37,38]
		LAMP	[86]
		gel-based PCR	[34,93]
Dengue and chikungunya	DBS, DSS	immunoenzymatic assay	[39,40,41,42,43,44]
	DBS	RT-PCR, qPCR	[90]
		gel-based PCR, RT-PCR	[96,97,98,99]
Foodborne trematodiases	DBS	immunoenzymatic assay	[45,46,47]
Human African trypanosomiasis	DBS	immunoenzymatic assay	[48,49,50,51,52]
		gel-based PCR	[100]
Leishmaniasis	DBS	immunoenzymatic assay	[8,53,54,55]
		LAMP	[87,88]
		qPCR	[53,87]
		gel-based PCR	[101,102,103,104,105]
Leprosy	DBS	immunoenzymatic assay	[56,57]
Lymphatic filariasis	DBS	immunoenzymatic assay	[58,59,60,61]
		gel-based PCR	[94,95]
Onchocerciasis	DBS	immunoenzymatic assay	[62,63,64]
		gel-based PCR	[62]
Schistosomiasis	DBS, DUS	immunoenzymatic assay	[65,66,67,81,82]
		gel-based PCR	[106,107,108,109,110]
	DUS	LAMP	[87]
Trachoma	DBS	immunoenzymatic assay	[68,69,70,71,72]
Yaws	DBS	immunoenzymatic assay	[73,74]
Taeniasis and cysticercosis	DBS, dried cerebrospinal fluid spot	immunoenzymatic assay	[75,76,77,78,83]
Soil-transmitted helminthiases	DBS	immunoenzymatic assay	[45,79,80]
Rabies	DBS	immunoenzymatic assay	[84,85]
		RT-PCR	[111]
	animal brain samples applied to filter paper	RT-hn-PCR	[112]

DBSs—dried blood spots; DSSs—dried saliva spots; DUSs—dried urine spots; RT-PCR—real-time polymerase chain reaction; qPCR—quantitative polymerase chain reaction; LAMP—loop-mediated isothermal amplification; RT-hn-PCR—real-time hemi-nested polymerase chain reaction.
